# Health disparities by occupation, modified by education: a cross-sectional population study

**DOI:** 10.1186/1471-2458-7-196

**Published:** 2007-08-08

**Authors:** Anita C Volkers, Gert P Westert, Francois G Schellevis

**Affiliations:** 1 NIVEL (Netherlands Institute for Health Services Research), Utrecht, The Netherlands; 2 RIVM (National Institute of Public Health and the Environment), Bilthoven/Tilburg University (TRANZO), Tilburg, The Netherlands

## Abstract

**Background:**

Socio-economic disparities in health status are frequently reported in research. By comparison with education and income, occupational status has been less extensively studied in relation to health status or the occurrence of specific chronic diseases. The aim of this study was to investigate health disparities in the working population based on occupational position and how they were modified by education.

**Methods:**

Our data were derived from the National Survey of General Practice that comprised 104 practices in the Netherlands. 136,189 working people aged 25–64 participated in the study. Occupational position was assessed by the International Socio-Economic Index of occupational position (ISEI). Health outcomes were self-perceived health status and physician-diagnosed diseases. Odds ratios were estimated using multivariate logistic regression analysis.

**Results:**

The lowest occupational position was observed to be associated with poor health in men (OR = 1.6, 95% CI 1,5 to 1.7) and women (OR = 1.3, 95% CI 1.2 to 1.4). The risk of poor health gradually decreased in relation to higher occupational positions. People with the lowest occupational positions were more likely to suffer from depression, diabetes, ischaemic heart disease, arthritis, muscle pain, neck and back pain and tension headache, in comparison to people with the highest occupational position (OR 1.2 to 1.6). A lower educational level induced an additional risk of poor health and disease. We found that gender modified the effects on poor health when both occupational position and education were combined in the analysis.

**Conclusion:**

A low occupational position was consistently associated working people with poor health and physician-diagnosed morbidity. However a low educational level was not. Occupational position and education had a combined effect on self-perceived health, which supports the recent call to improve the conceptual framework of health disparities.

## Background

Several mechanisms have been proposed to explain the relationship between a lower socio-economic status (SES) and an increased mortality and morbidity rate in the general population. [1-3] A lower socio-economic status influences health in an unfavourable way through the presence of unhealthy lifestyle factors, unequal access to – and quality of – health care, more material deprivation and a stressful psychosocial environment.[3] In contrast to education and income, occupation is a risk factor for poor health in itself, for example through, environmental risks such as to exposure to chemicals or adverse climatic conditions, ergonomic and physical demands, low skill discretion and a lower level of decision authority.[4,5] These influences are not incorporated in the earlier mentioned mechanisms. In Europe and the USA, health policies have reflected a renewed interest in socio-economic health inequalities. [6-8] This interest is generated by studies showing that socio-economic health disparities remained the same or even grew over the last decades. [9-12]

The three core dimensions of SES, educational level, occupational status and income, are strongly related and complementary, but not interchangeable.[13] Several European studies have reported associations between occupational status and different health outcome measurements. In the United States, education and income have most often been used. Disease specific mortality rates indicated that members of the manual class run a higher risk of dying from ischaemic heart disease, cancer and gastrointestinal diseases than those of the non-manual class.[14] Occupational class differences have also been found for several indicators of self-reported morbidity, for example, perceived general health, long-term disabilities and chronic conditions.[15] In Norway there was a clear gradient in the relationship between occupational class and self-reported ill health. Unskilled workers had more long-lasting illness limiting their capabilities, and perceived their health more often as less than good, compared to highly skilled non-manual workers.[16] In comparison to education and income, occupational position has been less extensively studied in relation to the occurrence of specific, chronic diseases in studies on health disparities. More insight into these relationships is required in order to find clues to shared and disease-specific pathways, and to account for the inverse social gradients that were recently found for some diseases such as allergy.[17] The finding that there are socio-economic differences in accurate reporting of diseases [18,19] underlines the need for physician-reported diagnoses in addition to self-reporting of health. In the main, studies on health disparities have focussed on the difference between manual and non-manual workers. Now that the number of non-manual workers is increasing, other occupational measures may be more meaningful and may reflect the social reality of today better.

For practical and cost reasons, studies on socio-economic health disparities often consider just one SES dimension. In cases where there is more than one dimension, they focus on the relationship between one SES indicator and health outcomes after adjustment for the other SES dimensions. The study of Snittker is one exception.[20] In this study the shape of the income-health gradient was examined by looking at the level of education. The positive relationship between income and health was found to vary both in its strength and shape by the level of education. For all levels of income those with more education had considerably better health. The income gradient flattened as education increases and the effect of education was greatest at lower levels of income. The findings were consistent for several aspects of self-reported health. Insight into the combined effect of SES indicators on health is relevant because of the recent call to improve the conceptual framework of health disparities [13].

The aim of this study was to investigate health disparities based on occupational position in the working population and how they were modified by education. We hypothesized that a lower occupational position is related to a poor health status and higher morbidity rates, independent of the level of education. People with both a lower occupational position and a lower educational level are more disadvantaged than expected, based on the individual effects of these two SES indicators. Data from the second Dutch National Survey of General Practice (DNSGP-2) were used, providing both information about self-perceived health, as well as diseases as diagnosed by general practitioners (GPs). Occupational position was assessed by the International Socio-Economic Index of occupational position (ISEI) which is characterized by a broad range of occupational positions on a hierarchical one-dimensional scale, instead of a distinction in occupational classes.

In this study two questions will be answered:

1) Is the occupational position a relevant SES indicator of health outcomes in addition to education?

3) Is there a combined effect of occupational position and educational level on health outcomes?

## Methods

### Data source

Our data were obtained from the second Dutch National Survey of General Practice (DNSGP-2).[21] The DNSGP-2 was conducted in 2001 in 104 general practices consisting of 195 GPs.

In the Netherlands all non-institutionalized inhabitants are listed with a general practice. Therefore, patients registered in Dutch general practice are representative of the general population in the Netherlands. The total practice population (n = 385,461) was representative, with respect to age, gender and type, of health care insurance – whether public or private – for all patients listed in Dutch general practices. This also implies that the total practice population is illustrative of the Dutch population in these regards. Subsequently, 294,999 patients from the total practice population (n = 385,461) responded in a socio-demographic census. This resulted in a response rate for all ages of 76.5%. Age and gender did not differ between responders and non-responders.

Among the responders to the socio-demographic census were 164.281 patients between 25–64 years. Subsequently, we selected all 136,189 patients with a valid value for occupational position based on their last occupation (82.9%). This was our original study sample. People within this study sample were referred to as working people. We have no reason to assume that patients in this study sample were different from the overall working population regarding their type of occupation, for example due to response bias. The remaining patients were without paid employment, such as students, housewives and pensioners (12.4%), or had a missing value for their occupational position (5.7%). The privacy of the participating patients was guaranteed according to the Dutch legislation.

### SES indicators

The socio-demographic census was a one page questionnaire sent by mail before the start of the DNSGP-2. Participants were asked to fill in their last occupation, which was coded using the Dutch Standard Classification of Occupations of Statistics Netherlands [22], which is strongly related to the International Standard Classification of Occupation (ISCO88).[23] This code was transformed to a score on the International Socio-Economic Index of Occupational Status (ISEI).[24,25] The construct of this index is based on the assumption that occupation is an intervening mechanism between education and income. The range of positions on the ISEI scale is 16–87 and positions are derived from the average educational level and income related to that occupation. Information about education was obtained by asking about educational attainment, which was classified in the following way: none, elementary school, high school and college or university. This classification was simplified as low, middle and high educational level.

### Health outcomes

Self-perceived health was assessed by one single question in the socio-demographic census. There were 128,730 patients from the original study sample who answered the question about health status. Respondents described their general health status as: 1) "very good", 2) "good", 3) "neither good nor poor, 4) "poor" and 5) "very poor". We dichotomized this variable in fair health (first two categories) and poor health (remaining categories). Diagnostic information was derived from the electronic medical records of the patients kept by GPs in the practice computer during the one-year registration. All GPs in this study diagnosed complaints and diseases according to the International Classification of Primary Care-1 (ICPC), which is an international standard diagnostic classification system accepted by the WHO.[26] Eight practices were omitted due to incomplete data concerning 7513 people. These practices recorded less than 6 months, had on average less than 5 doctor-patient contacts per listed patient per year, or recorded in more than 50% of the doctor-patient contacts no ICPC diagnoses. Morbidity data were therefore available for 128,676 patients from the original study sample. Excluding patients from the study sample did not influence the distribution of quartile groups of occupational position, educational level, age, gender and health insurance. We selected 8 diseases with a SES gradient [17,27,28]: depression (ICPC code: P76), diabetes (ICPC code: T90), ischaemic heart disease (ICPC codes: K74, K75 and K76), osteoarthritis (ICPC codes: L89, L90 and L91), dermatitis (ICPC code: S88), muscle pain (ICPC codes: L01 till L03 and L83), neck and back pain (ICPC code: L86) and tension headache (N02). The outcome measure was the presence or absence of each disease.

### Analysis

The study sample was divided into quartiles based on their ISEI score in order to study the social gradient as well as to provide sufficient power for the statistical analysis by limiting the number of interaction terms. Multivariate logistic regression analysis was used to test, simultaneously, the relationship between occupational position and health outcomes and the relationship between educational level and health outcomes while controlling for age. The sample sizes used in the statistical analyses with self-perceived health and morbidity were 128,730 and 128,676, respectively (see section health outcomes). Outcome variables were poor, versus fair, health status (1 versus 0) and the presence, versus absence, of each one of the diseases (1 versus 0). The highest quartile group of occupational position and people with a high educational level were taken as a reference. All odds ratios in the regression analysis were adjusted for age and analyses were conducted separately for men and women. Subsequently, the models were extended with the interaction terms between occupational position and educational level in order to test the combined effect of occupational position and educational level on the health outcomes, or in other words to test modification effects. Table 1 shows the terms of the simple and extended model. In case there was an improved model fit (comparison between the -2 log likelihood of both models) and there were significant interaction terms, the relationship between occupational position and health outcomes was further explored by stratification to educational level.

**Table 1:** Effect terms in the multivariate logistic regression analysis

Effects model 1	Effects model 2
Main terms:	Main terms:
Occupational position	Occupational position
lowest quartile	lowest quartile
second quartile	second quartile
third quartile	third quartile
highest quartile (reference)	highest quartile (reference)
	
Educational level	Educational level
low	Low
middle	middle
high (reference)	high (reference)
	
Age	Interaction terms
	low educational level × lowest quartile of occupational position
	low educational level × second quartile of occupational position
	low educational level × third quartile of occupational position
	middle educational level × lowest quartile of occupational position
	middle educational level × second quartile of occupational position
	middle educational level × third quartile of occupational position
	high educational level × highest quartile of occupational position (reference)
	
	Age

Analyses were carried out using SPSS version 11.5 for windows. The significance of odds ratios was tested by 95% confidence intervals.

## Results

**Figure 1:**
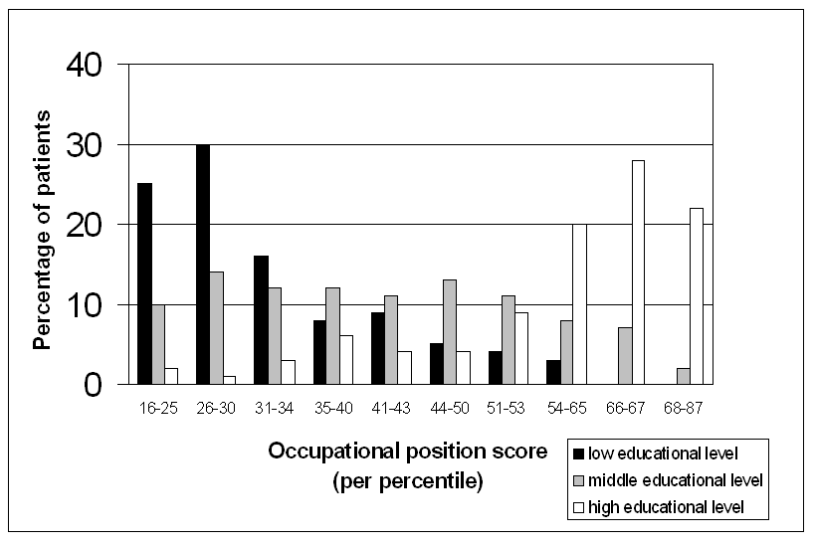
Distribution of educational level by occupational position.

Table 2 shows the characteristics of the total study sample and of each quartile group of occupational position. There were slightly more men than women, with the exception of the third quartile group. The age distribution of the total study population showed a large degree of similarity with that of each quartile group. A low educational level occurred more often in people with a low occupational position and a high educational level in people with a high occupational position. The majority, however, had an average, or middle, level of education (Figure 1). 16.7% of the working people had a poor self-perceived health status and there was a gradual decline towards a higher occupational position from 21.9% to 12.4%. In cases of depression, diabetes, osteoarthritis, dermatitis, and muscle pain, prevalence was highest in people with the lowest occupational position and lowest in people with the highest occupational position. There was a clear social gradient in prevalence of diabetes, osteoarthritis and muscle pain.

**Table 2:** Description of demographic variables, educational level and health outcomes by occupational position

		Occupational position			
	Total group	Lowest quartile score 16–32	Second quartile score 33–43	Third quartile score 44–58	Highest quartile score 59–87

Gender (/100)^a^					
Male	52.5	53.7	53.0	41.5	61.2
Female	47.5	46.3	47.0	58.5	38.8
					
Age group (/100)^a^					
25–44 year	55.3	53.2	57.1	57.3	53.8
45–64 year	44.7	46.8	42.9	42.7	46.2
					
Educational level (/100)^a^					
low (none/elementary)	8.7	21.3	9.6	3.8	0.7
middle (high school)	63.8	73.2	77.8	73.1	32.6
high (college/university)	27.5	5.5	12.7	23.1	66.7
					
Self-perceived health status(/100)^b^					
Poor	16.7	21.9	17.3	15.5	12.4
Fair	83.3	78.1	82.7	84.5	87.6
					
Prevalence morbidity (/1000)^c^					
Depression	27	32	25	28	22
Diabetes	18	27	20	17	14
Ischaemic heart disease	9	15	11	9	9
Osteoarthritis	9	11	10	8	7
Dermatitis	47	52	47	49	42
Muscle pain	18	24	18	16	13
Neck and back pain	13	16	12	11	13
Tension headache	9	10	8	9	8

^a ^percentages based on the original study sample (n = 136,189), ^b ^percentages based on people who reported their health status (n = 128,730), ^c ^percentages based on people with valid morbidity data (n = 128,676)

Among men, occupational position had a strong and significant effect on their self-perception of poor health, independent of educational level (Table 3). The odds ratios decreased from the lowest quartile group (1.59, 95% CI 1.49 to 1.71) to the third quartile group (1.19, 95% CI 1.11 to 1.28). So, men with a lower occupational position were more likely to report poor health. Occupational position was also related to the prevalence of most of the diseases, with the exception of dermatitis and tension headache. The highest odds ratios were found for osteoarthritis, muscle pain and neck and back pain. The effect of occupational position was mainly found in the lowest quartile group of occupational position. Educational level had a strong effect on self-perceived health status, but its effect on morbidity was more disease specific than that of occupational position.

**Table 3:** Odds ratios (95% CI) of poor health and morbidity by occupation and education (age adjusted)

		Presence of morbidity							
	Poor health status	Depression	Diabetes mellitus	Ischaemic heart disease	Osteoarthritis	Dermatitis	Muscle pain	Neck and back pain	Tension headache

Men:									
									
Occupational position									
Lowest quartile	1.59 (1.49–1.71)	1.38 (1.15–1.66)	1.24 (1.05–1.47)	1.34 (1.10–1.63)	1.45 (1.09–1.95)	1.09 (0.96–1.24)	1.49 (1.22–1.82)	1.42 (1.32–1.53)	1.17 (0.88–1.56)
Second quartile	1.36 (1.27–1.45)	1.08 (0.90–1.30)	1.16 (0.98–1.37)	1.14 (0.94–1.39)	1.46 (1.10–1.93)	1.14 (1.01–1.28)	1.12 (0.91–1.36)	1.15 (1.07–1.24)	0.90 (0.67–1.21)
third quartile	1.19 (1.11–1.28)	1.19 (0.99–1.41)	1.18 (1.00–1.40)	1.17 (0.96–1.42)	1.17 (0.87–1.57)	1.00 (0.89–1.14)	1.01 (0.82–1.25)	1.00 (0.93–1.08)	1.11 (0.84–1.47)
highest quartile	1	1	1	1	1	1	1	1	1
									
Educational level									
low	2.35 (2.15–2.56)	1.10 (0.87–1.38)	2.06 (1.69–2.51)	1.46 (1.15–1.85)	1.09 (0.77–1.56)	1.14 (0.97–1.35)	1.47 (1.13–1.91)	1.44 (1.30–1.59)	1.10 (0.76–1.60)
middle	1.35 (1.27–1.43)	0.96 (0.83–1.13)	1.23 (1.06–1.44)	1.26 (1.05–1.51)	1.17 (0.90–1.52)	1.04 (0.93–1.15)	1.34 (1.12–1.62)	1.39 (1.30–1.49)	0.90 (0.70–1.15)
high	1	1	1	1	1	1	1	1	1
									
Women:									
									
Occupational position									
lowest quartile	1.31 (1.21–1.41)	1.27 (1.09–1.48)	1.33 (1.16–1.53)	1.55 (1.01–2.38)	1.26 (0.94–1.68)	1.15 (1.02–1.30)	1.42 (1.15–1.75)	1.22 (1.13–1.33)	1.21 (0.92–1.59)
second quartile	1.10 (1.02–1.89)	1.09 (0.92–1.24)	1.12 (0.97–1.29)	1.10 (0.71–1.70)	1.14 (0.86–1.52)	0.94 (0.83–1.06)	1,11 (0.90–1.37)	1.10 (1.02–1.19)	1.00 (0.76–1.31)
third quartile	1.06 (0.99–1.14)	1.11 (0.97–1.28)	1.03 (0.90–1.19)	1.11 (0.72–1.68)	1.03 (0.78–1.35)	1.07 (0.96–1.20)	1.07 (0.88–1.31)	1.04 (0.96–1.12)	1.04 (0.81–1.34)
highest quartile	1	1	1	1	1	1	1	1	1
									
Educational level									
low	2.27 (2.01–2.49)	1.10 (0.99–1.44)	2.30 (1.96–2.71)	1.35 (0.83–2.18)	1.23 (0.89–1.70)	1.24 (1.06–1.45)	2.08 (1.62–2.65)	1.49 (1.13–1.33)	1.33 (0.96–1.86)
middle	1.19 (1.11–1.26)	1.02 (0.90–1.15)	1.36 (1.19–1.55)	1.25 (0.84–1.84)	1.12 (0.87–1.45)	1.16 (1.05–1.28)	1.50 (1.25–1.79)	1.31 (1.22–1.40)	1.07 (0.86–1.34)
high	1	1	1	1	1	1	1	1	1

In women the relationship between occupational position and self-perceived health was less pronounced than in men. Both the lowest and second quartile groups were more likely to report poor health in comparison to the highest quartile group (OR 1.31, 95% CI 1.21 to 1.41 and OR 1.10, CI 1.02–1.89 respectively). Occupational position had the largest effect on ischaemic heart disease and muscle pain. Women with the lowest occupational position were also more likely to have depression, diabetes and neck and muscle pain. Just as in men, educational level had a strong effect on self-perceived health and the prevalence of some of the diseases.

### Modification effect

In the following step, the effect of modifying the relationships between occupational position and health measurements by educational level was tested in men. The fit of the extended model was significantly improved in comparison to the simple model (χ^2 ^= 29, df = 6, p < 0.001). We found an odds ratio of 1.21 (95% CI 1.00 to 1.45) for the interaction term, middle educational level × second quartile group of occupational position, and an odds ratio of 1.21 (95% CI 1.00 to 1.45) for the interaction term, middle educational level × third quartile group of occupational position. To interpret these combined effects of occupational position and education, stratified analyses were performed (Table 4). Among people with a low educational level, occupational position had strong relationships with poor health, irrespective of the quartile group. Significant odds ratios were also observed in the middle educational group and reduced gradually in higher quartile groups. In the high educational group, the increased risk of the lowest occupational position on poor health remained present, but disappeared in the other quartile groups. Educational level did not modify any of the relationships between occupational position and specific diseases as diagnosed by the GP.

**Table 4:** Odds ratios (95% CI) of poor health stratified to educational level (age adjusted)

	Health status	
	Men	Women

Low educational level with occupational position:		
lowest quartile	1.67 (1.09–2.54)	1.14 (0.71–1.83)
second quartile	1.74 (1.13–2.68)	1.04 (0.64–1.68)
third quartile	1.67 (1.06–2.63)	0.97 (0.59–1.59)
highest quartile	1	1
		
Middle educational level with occupational position:		
lowest quartile	1.71 (1.58–1.86)	1.36 (1.22–1.51)
second quartile	1.40 (1.28–1.52)	1.15 (1.04–1.28)
third quartile	1.25 (1.14–1.37)	1.09 (0.98–1.20)
highest quartile	1	1
		
High educational level with occupational position:		
lowest quartile	1.94 (1.58–2.39)	1.38 (1.13–1.67)
second quartile	1.18 (1.00–1.40)	0.97 (0.84–1.12)
third quartile	1.07 (0.94–1.21)	1.10 (0.99–1.23)
highest quartile	1	1

In women the fit of the extended model was also improved, although not significantly (χ^2 ^= 7, df = 6). The relationship between occupational position and health status was modified by education, which was indicated by the significant interaction term of the middle educational level × second quartile group (OR 1.21, 95% CI 1.01 to 1.44). Stratification shows that occupational position had no effect on poor health in women with a low educational level. Women with a middle educational level had an increased risk of poor health if they belonged to the lowest and second quartile group of occupational position. In women with a high educational level there was only an effect of occupational position in the lowest quartile group. The extended model for depression showed an insignificant improved model fit (χ^2 ^= 10, df = 6) with significant, reversed interaction terms. These are the low educational level × lowest quartile group (OR 0.38, 95% CI 0.16 to 0.86) and low educational level × second quartile group (OR 0.32, 95% CI 0.14 to 0.72). In the stratified analyses this resulted in a reduced risk of depression in women with a low educational level within the second quartile group and, on the contrary, an increased risk of depression in women with a middle and high educational level within the lowest quartile group.

## Discussion

This study demonstrated disparities in self-perceived health and physician-reported morbidity between working people (25–64 year) with lower and higher occupational positions. A lower occupational position was related to poor health and a variety of diseases such as depression, diabetes, ischaemic heart disease, muscle pain and neck and back pain. These relationships were independent of educational level that, in itself, had a strong effect on poor health and some of the diseases. Occupational position played a more consistent role in socio-economic differences in specific diseases, than did education. There was a social gradient in the risk of poor health by occupational position in men and women, while the effect on specific diseases was mainly restricted to differences between people with the lowest and highest occupational position. Educational level modified the effect of occupational position on health status, but not on GP-reported morbidity. The combined effects were gender specific.

Socio-economic disparities in self-reported chronic diseases [17] show many similarities with our findings of socio-economic disparities in physician-diagnosed diseases. However, we found no inverse relationship between occupational position and one of the selected diseases. Our findings show that occupation is indeed a relevant SES indicator of health disparities and emphasizes the need of more than one SES indicator in studies on socio-economic disparities in health. In 1982 Abramson et al. had already argued that there might be considerable gains from using more than one SES indicator to understand the social class relationship of health characteristics.[29] Recently, Braveman et al. also recommended measuring as much relevant socio-economic information as possible.[13] Such SES indicators as occupation, education and income are complementary and not just interchangeable. Vom den Knesebeck et al. concluded that income is the 'best' SES indicator of health disparities.[3] In our opinion it is not just a matter of finding the 'best' SES indicator that explains most of the variance in health outcomes. Insight into the diversity of the relationships between SES indicators and different health outcomes improves the description of socio-economic health disparities and thereby the conceptual framework of health disparities. This is in contrast with the choice, for practical reasons, of one SES indicator in most epidemiological studies.

The findings did not confirm the hypothesis that people with the lowest occupational position and a low educational level had a multiplicative risk of poor health and the prevalence of diseases. This hypothesis was generated by a previous finding that a lower education had a larger unfavourable effect on health at lower levels of income.[20] In men with a low educational level, the increased risk of poor health was similar, irrespective of occupational position. In female workers, we observed no significant effect of occupational position when women had a low educational level. However, we found for men and women that differences in poor health status by occupational position were most pronounced in highly educated people. The combined effects of SES indicators on health status should be incorporated in future studies on health disparities and may result in a better understanding of the underlying mechanisms of socio-economic differences in health.

Reducing health disparities is an important goal of public health, but programmes do not focus on low occupational groups.[8,9,30] Occupation holds an intermediate position between education and income and is an important determinant of health in its own right through health risks related to employment. Collaboration between policy makers in public health and occupational health may be inevitable to reduce occupation-related health disparities and therefore socio-economic health disparities in general. Health policy makers should consider available information about the combined effect SES indicators of health disparities in order to optimize their strategies.

### Limitations

In this study we asked people about their last occupation instead of their current occupation. Therefore, our study sample also included people who were, temporarily at least, unemployed. We found a valid occupational status in about 83% of the people in our study, whereas the employment rate among people aged 25–64 years in the Netherlands was about 70% at that time. Occupational position measured by the ISEI is assumed to be an intervening factor between education and income.[24,25] We can not rule out that the independence of occupational position and educational level may have influenced the modifying role of education in this study. Nevertheless, both socio-economic factors had strong and individual effects on health status and morbidity. The purpose of this study was not explicitly to unravel the mechanisms underlying the relationships with specific diseases, although the findings may contribute to understanding socio-economic health disparities. For instance, the role of lifestyle factors, access to, and quality of, health care, material deprivation, psychosocial environment and working conditions were not taken into account. A shortcoming of the study is the lack of a measure of income for the entire study population, while we emphasized the importance of a complete set of SES indicators. The cross-sectional design of the study limits the determination of causality of relationships and the influence of an effect on health selection.

## Conclusion

In conclusion, occupational position is a relevant SES indicator in research on health disparities among the working population. A low occupational position was consistently associated with poor health and physician-diagnosed morbidity, which could not be explained by a low educational level. The social gradient was most prominent in the risk of poor health by occupational position in men and women. In addition, occupational position and education had a combined effect on self-perceived health which was gender specific. Our findings did not confirm the hypothesis that people with the lowest occupational position and a low educational level had a multiplicative risk of poor health and prevalence of disease. On the other hand, we found for men and women that differences in poor health status by occupational position were most pronounced in highly educated people. To improve the description of socio-economic health disparities, and thereby the conceptual framework of health disparities, future research should focus on the role of occupational status in addition to education and income and incorporate the combined effects between all the SES indicators. Health programmes on health disparities should also focus on low occupational groups.

## Competing interests

The author(s) declare that they have no competing interests.

## Authors' contributions

All authors were responsible for the conception and design of the study, revision and approval of the paper. AV interpreted the data, performed the statistical analysis and drafted the paper. GW and FS helped to draft the final manuscript.

## Pre-publication history

The pre-publication history for this paper can be accessed here:


